# Increasing family planning in Myanmar: the role of the private sector and social franchise programs

**DOI:** 10.1186/s12905-017-0400-4

**Published:** 2017-07-01

**Authors:** Tin Aung, Nang Mo Hom, May Sudhinaraset

**Affiliations:** 1 Population Services International, Myanmar, No. 16, Shwe Gon Taing Street 4, Yangon, Myanmar; 2 Global Health Sciences, Department of Epidemiology and Biostatistics, University of California, San Francisco, 550 16th Street, San Francisco, CA 94158 USA; 3 Community Health Sciences, University of California, Los Angeles, 650 Charles E Young Dr. S, 21-245C, Los Angeles, CA 90095 USA

**Keywords:** Myanmar, Family planning, Social franchise program, Private sector

## Abstract

**Background:**

This study examines the influence of clinical social franchise program on modern contraceptive use.

**Methods:**

This was a cross-sectional survey of contraceptive use among 2390 currently married women across 25 townships in Myanmar in 2014. Social franchise program measures were from programmatic records.

**Results:**

Multivariable models show that women who lived in communities with at least 1-5 years of a clinical social franchise intrauterine device (IUD) program had 4.770 higher odds of using a modern contraceptive method compared to women living in communities with no IUD program [CI: 3.739-6.084]. Townships where the reproductive health program had existed for at least 10 years had 1.428 higher odds of reporting modern method use compared to women living in townships where the programs had existed for less than 10 years [CI: 1.016-2.008].

**Conclusions:**

This study found consistent and robust evidence for an increase in family planning methods over program duration as well as intensity of social franchise programs.

## Background

With a total population of 51.4 million people, Myanmar continues to lag behind other countries in the region across multiple maternal and child health indicators [1]. While use of family planning in Myanmar has steadily increased over the past two decades, contraceptive prevalence rates remain low [2, 3]. The most recent nationally representative data in Myanmar, the Multiple Indicator Survey Cluster (MICS) conducted in 2009-2010, suggests that only 46.0% of ever-married women used any method of modern contraception. The most common methods were injectables (27.5%), followed by contraceptive pills (11.5%), and intrauterine devices (IUDs) (2.1%) [2]. Data also suggests that there are significant rural and urban disparities, with 49% of urban and 34% of rural women using a form of modern contraception [3]. This reflects the greater availability and ease of access to modern methods in urban areas compared to rural settings.

Historically in Myanmar, reproductive health (RH) services have been delivered through the public health sector in both urban and rural areas [4]. However, only 2.3% of GDP is spent on healthcare [5]. Similar to other Asian countries, out-of-pocket expenditure for healthcare (percentage of private expenditure for health) is high. Myanmar reports one of the highest in the world, at 93.7% of total health expenditure [5]. Thus, the private sector healthcare system has become an essential part of the health system. The public health sector consists of multiple levels of hospitals, urban and rural health centers, and a number of voluntary community based workers known as Auxiliary Midwives (AMWs), which are primarily attached to rural health centers. In urban and rural areas, there are also a variety of private general practitioners (GPs), private clinics, and drug shops where women can purchase contraceptive services and supplies.

Currently, the majority of modern family planning methods are delivered through the private sector (51.8%) compared to the public sector (42.4%); however, the large majority of long-term methods are provided by the public sector [3]. Long term methods such as intrauterine devices (IUDs), implants and permanent methods such as male and female sterilization are typically not offered in the private sector. However, recent studies suggest that women prefer the private sector and non-governmental organizations (NGOs) because of perceived higher quality and perceptions of less judgment when attending private clinics [6]. Few programs exist in Myanmar that engages the private sector at scale.

Population Services International/Myanmar (PSI/M) is one of the key organizations that provide reproductive and family health care services in the private sector in Myanmar through clinical social franchising. Globally, clinical social franchising has gained popularity in the past two decades as one approach to engaging the private sector [7]. A clinical social franchise program is a type of health service delivery model that networks existing private providers in order to increase accessibility to low-priced, high quality products and services. This results in a uniform and standardized set of services to improve availability and expertise in a variety of health services, including sexual and reproductive health services, child health, tuberculosis (TB) treatment, malaria testing and treatment, and HIV care. Social franchise programs have shown to be cost-effective [8], reach the poorest populations, particularly in urban settings [9], improve malaria knowledge and practices [10], increase TB treatment [11], and improve clients’ perceived quality [4].

While studies have documented that social franchise programs improve facility-level outcomes, less is known about how franchise programs influence women’s contraceptive use. The few studies that do exist highlight the promise of social franchise programs in family planning use. One study found that social franchise clinics increased monthly reproductive health client volume in Myanmar, the number of family planning brands available, and had somewhat higher client satisfaction [12]. In Vietnam, a prospective study found that joining a social franchise increased overall client volume by 40%, and specifically increased reproductive health services by 51%. However, the increase in client volume was not associated with expanded health access at the community level or increase in reported clinic use [13]. Most studies have focused on clinic or facility-level data; household level data, measuring women’s contraceptive use, is needed to understand how social franchise programs influence contraceptive use.

The objective of this study was to examine the influence of clinical social franchise program on modern contraceptive use. This study is novel because it assesses the influence of social franchise programs across 25 townships. Because of the stepped-wise approach of introduction of reproductive health and IUD programs across townships to franchised clinics, this study is able to assess the impact of duration of program as well as intensity of the programs on modern contraceptive use.

## Methods

### Setting and description of program

Mynamar offers a unique setting to assess the impact of clinical social franchising on reproductive health and family planning. In 2001, Population Services International (PSI) Myanmar started a social franchising network by recruiting general practitioners (GPs) branded as the Sun Quality Health (SQH) clinics (for more information on SQH franchise, see for example [9, 11, 12]). PSI expanded its network gradually in phases both in geographical coverage and types of services it supports. At the time of the present study in 2014 it included 1268 clinics, and provided reproductive health (RH) services in 208 out of 324 townships of Myanmar. PSI/M implemented the RH program first in its franchise network of clinics. When a clinic joined the RH program, PSI provided training on RH services in general including short-term hormonal family planning methods such as daily pills and 3-months hormonal injections. The training also included counseling and how to increase client’s method choice. PSI also distributed these products to the franchise clinic at a highly subsidized rate, and produced and made available educational pamphlets and posters at these clinics.

In 2004, PSI/M introduced the IUD program to those clinics that were already part of the RH program. IUD insertion training was conducted with doctors that were part of he RH program. The training covered counseling, infection prevention, IUD insertion and management of complication and side effects. Trained doctors also participated in practical session on IUD insertion of live patients after the training. Their performance and skills were monitored through supportive supervision visits by a training team as well as quality assurance visits by a quality assurance team of PSI/M until they could perform the IUD insertion independently. PSI/M provided IUD products at a highly subsidized rate. As part of this program, PSI also used RH promoters to build awareness on family planning through community health talks followed by the distribution of referral cards to eligible women to go to a SQH clinic for family planning services. RH promoters are trained and employed by PSI/M. At the time of the survey, 116 clinics in 93 townships had joined the IUD program.

### Study design and data collection

A cross-sectional study with a multistage cluster sampling approach was conducted among 2390 currently married women to assess contraceptive use. Only currently married women were recruited for our household survey because the majority of PSI’s clients who attend clinical social franchise clinics for family planning are married. Married and unmarried women would have different determinants and therefore only married women were recruited in this sample. A total of 25 program townships were included to allow for sufficient power and precision to detect contraceptive use among the representative sample of women. At the first stage, using proportionate to population size method (PPS), we selected 25 townships out of 93 townships where at least one Sun Quality Health (SQH) clinic provided family planning services. Because the selected sample size was 2390 women across selected 25 townships we selected 95 respondents from each township. In Myanmar 30% of the total population live in urban areas and the other 70% in rural areas on average the sample distribution is 30% in urban and 70% in rural areas that yielded 734 respondents and 1656 respondents from urban and rural areas respectively. A total of 29 respondents from urban and 66 from rural were recruited for each selected township.

The second stage involved selecting wards in urban areas and village tracts in rural areas of the selected townships. The number of wards varies by township. Population sizes are not available at ward and village tract level and the number of wards varied from two to 39 wards in selected townships. We used systematic random sampling method for sampling respondents. For rural areas, the number of village tracts did not vary as much as in wards, and an equal number of six villages were randomly selected in each township. Systematic random sampling was used, with 11 households selected from each village.

On arriving at the selected ward/village, interviewers drew a rough map of the ward/village and identified the households (by door to door visits to each house) where the women currently married within the ages of 18 and 49 years (potential respondents) were living because the list of all households and households with currently married women was not available. Using the rough map and systematic random sampling we selected 29 households in a ward and 11 households in a village. We interviewed only one currently married woman fitting the age requirement.

The survey covered a number of demographic characteristics and health outcomes, including household roster, socio-economic status, contraceptive history, fertility preferences, ability and motivation factors related to the use of contraceptives, and source of family planning method. In addition, social franchise program measures were derived from programmatic records and measured at a township level.

Verbal informed consent was collected. Verbal consent, rather than written consent, was used because these areas have high illiteracy rates and therefore verbal consent is more appropriate. Verbal consent was also used to reduce the risks associated with potential breach of confidentiality.

### Measures

The main outcome of interest is use of modern family planning method. This measure was generated using a series of questions in the survey of current method that women used. Women were categorized as using a modern method of contraception if they indicated that they used IUDs, injectables, male or female condoms, pills, female sterilization, or implants. To assess social franchise program measures, township level data was included. First, the study assessed the association between duration of the reproductive health program and family planning use. The duration of reproductive health program is defined as the difference between the survey year (2014) and the introduction of the program. The duration of the IUD program was constructed in a similar manner. To assess the intensity of the program, the study included data on the number of reproductive health providers that were franchised as well as the number of IUD providers that were franchised in each township. Because these four measures are collinear (i.e. the longer the program has been in existence, the probability that the number of providers has increased), each program measure was modeled separately with the outcome data.

The study also included a variable on source of method, and includes public, private, and other sources. “Public sector” included government hospitals, health centers, government mobile clinics, and government field workers. “Private sector” included private hospitals, private clinics, pharmacies, private mobile clinics, franchised clinics, field workers, and grocery stores. “Other sources” included friend and relatives.

Demographic characteristics were also included in the models, including urban/rural residency, age categories, education level of the participant, parity and religion.

### Analyses

All analyses were carried out in STATA 12MP. Three sets of analyses were conducted. First, bivariate analyses, including chi-2 for categorical variables and t-tests for continuous variables, were used to assess differences across outcomes. Next, the study conducted a population average model analysis accounting for clustering at the township level. Township level variables included social franchise program characteristics such as duration of the program and number of providers. Finally, multivariable logistic regression was used to test the associations between program characteristics and modern contraceptive use, accounting for differences in demographic characteristics across townships.

## Results

### Demographic characteristics

Table 1 summarizes the demographic characteristics of study participants. In total, there were 2390 currently married women included in the study. The table stratifies the data into townships where franchised clinics have provided RH services for less than 10 years (*n* = 1320) and 10 or more years (*n* = 1070).

**Table 1:** Demographic characteristics of the study population stratified by duration of family planning program in the townships

	Less than 10 years (*N* = 1320; 55.2%)	10 or more years (*N* = 1070; 44.8%)	Total (*N* = 2390; 100%)	*p*-value
Residency				
Urban residency	29.1%	32.7%	30.7%	0.056
Rural residency	70.9%	67.3%	69.3%	
Age of respondent				
18-24	9.7%	10.1%	9.9%	0.493
25-34	42.9%	40.5%	42%	
35-49	47.4%	49.4%	48.3%	
Education level				
No formal education	12.0%	8.8%	10.6%	0.135
Some primary school	33.7%	33.8%	33.8%	
Middle school	28.9%	30.4%	29.6%	
High school	18.0%	19.3%	18.5%	
Graduate	7.3%	7.8%	7.5%	
Parity				
0 children alive	5.6%	3.4%	4.6%	0.006
1 child	29.2%	33.0%	30.9%	
2 children	29.0%	29.3%	29.1%	
3 children	19.2%	16.9%	18.2%	
4 children	8.7%	7.0%	7.9%	
5 or more children	8.3%	10.5%	9.2%	
Religion				
Buddhist	90.6%	97.9%	93.8%	0.000
Number of RH providers				
1-5 providers	53.9%	9.5%	34.1%	0.000
6-12 providers	38.4%	31.0%	35.1%	
13+ providers	7.7%	59.4%	30.8%	
Number of IUD providers				
0 IUD	15.5%	0.0%	8.6%	0.000
1 IUD provider	53.8%	62.2%	57.6%	
2+ IUD providers	30.7%	37.8%	33.8%	

Across these two groups, the study found that there were no statistically significant differences across urban/rural residency (29.1 vs. 32.7%; *p* = 0.056). Similarly, the age categories and education achievement of participating women were not different between the areas (*p* < 0.05). However, there were slight differences in parity across the two groups. We found that the proportion of women with three or four live children were slightly higher in the less-than-10-years serviced townships. The proportion of women with 5 or more live children was higher in the 10- or-more-years serviced townships (8.3 vs. 10.5%; *p* = 0.006). Women in the study were mostly Buddhists (93.8%); however, the proportion of women in 10- or-more-years serviced townships who were Buddhists was slightly but significantly higher than the proportion of women in less-than-10-years serviced townships (97.9 vs. 90.6%, *p* = 0.000).

At the township level, we found that there were more RH or IUD service providers in longer serviced townships. Women from 10-or-more-years serviced townships were likely to be serviced by 13 or more RH providers (59.4 vs. 7.7%; *p* = 0.000) and similarly by 1 (62.2 vs. 53.8%) or more than one IUD providers (37.8 vs. 30.7%; *p* = 0.000).

### Method mix by reproductive health service program duration

Across 25 program townships, approximately 60% of women used some form of modern method. In total, injectables were the most common method of contraception, with 37.4% of women reporting using this form of method [see Table 2]. Next, 13.3% of women used pills, 3.7% used an IUD, and 1.2% used condoms. Additionally, 4.4% of women used another form of modern method, including sterilization and female condoms. Women living in townships with 10 or more years of the reproductive health service program had higher prevalence of IUD use, injectables, pills, condoms, and other forms of contraception. Overall, women living in townships with 10 or more years of the program also reported higher modern method use. *P*-values were not statistically significant; therefore, we do not present them in the table.

**Table 2:** Method mix for townships with reproductive health program for less than 10 years and 10 years or more

	Less than 10 years (%) (*n* = 1320)	10 or more years (%) (*n* = 1070)	Total (%) (*n* = 2390)
IUD	3.2	4.4	3.7
Injectables	34.8	40.7	37.4
Pills	12.6	14.1	13.3
Condoms	1.1	1.3	1.2
Other	4.4	4.4	4.4
No method used	43.9	35.0	40.0

### Source of modern method

Table 3 shows the source of contraceptive method, by public and private sector. Among the users for all modern methods combined 40.3% of women received their methods from private sector including the franchised clinics. Other sources of family planning methods (i.e. friends/relatives) accounted for only 0.5%. Women were more likely to receive pills and condoms from private than the public sector (84.2 vs, 15.5% for pills and 93.1 vs. 6.9% for condoms). Compared to the private sector, the public sector were more likely to provide injectables (76 vs. 23.5%), IUD (56.2 vs. 43.8%) and other methods such as women sterilization (65.7 vs. 33.3%).

**Table 3:** Sources of modern contraceptives as reported by the respondents

	Inject (%)	Pills (%)	IUD (%)	Condoms (%)	Other: sterilization, female condom, etc. (%)	Total (%)
Public (*n* = 850)	76.0	15.5	56.2	6.9	65.7	59.2
Private (*n* = 578)	23.5	84.2	43.8	93.1	33.3	40.3
Others (*n* = 7)	0.6	0.3	0	0.0	1.0	0.5
Total (*N* = 1435)	100	100	100	100	100	100

### Multivariable models: Social franchise program characteristics and use of modern methods

Four separate multivariable models are included in Table 4. Each model controlled for township-level effects as well as demographic characteristics, including urban/rural residence, age, education level, parity, and religion. In model 1, the main predictor of interest was duration of the IUD program. Women who lived in communities with at least 1-5 years had 4.770 higher odds of using a modern contraceptive compared to women living in communities with no IUD program [CI: 3.739-6.084]. Similarly, women in communities with six or more years of IUD program introduction were 5.170 times more likely to use a modern method compared to programs without IUD introduction [CI: 4.048-6.603]. In this model, living in an urban area, being younger, higher education level, and being Buddhist was associated with higher contraceptive use.

**Table 4:** Program characteristics and use of modern methods

	(1)	(2)	(3)	(4)
Duration of IUD Program				
0 year (Reference)	1.0			
1-5 years	4.770***			
	(3.739 - 6.084)			
6+ years	5.170***			
	(4.048 - 6.603)			
Duration of RH Program				
< 10 years (Reference)		1.0		
10+ years		1.428**		
		(1.016 - 2.008)		
Number of PSI IUD Providers				
0 providers (Reference)			1.0	
1 provider			3.647***	
			(2.076 - 6.406)	
2+ providers			2.667***	
			(1.520 - 4.681)	
Number of PSI RH Providers				
1-5 providers (Reference)				1.0
6-12 providers				1.519*
				(0.972 - 2.375)
13+ providers				1.458*
				(0.945 - 2.249)
Urban	1.182*	1.137	1.161	1.152
	(0.972 - 1.438)	(0.924 - 1.398)	(0.955 - 1.410)	(0.936 - 1.418)
Age categories (18-24 reference)				
25-34 years	0.561***	0.632*	0.546***	0.625*
	(0.367 - 0.858)	(0.379 - 1.051)	(0.355 - 0.839)	(0.379 - 1.030)
35-49 years	0.333***	0.390***	0.329***	0.385***
	(0.213 - 0.520)	(0.221 - 0.688)	(0.212 - 0.512)	(0.222 - 0.665)
Education level (no education reference)				
Some primary education	1.289*	1.594*	1.368**	1.594**
	(0.972 - 1.709)	(0.999 - 2.544)	(1.019 - 1.837)	(1.028 - 2.471)
Middle school	1.297	1.611*	1.367	1.590*
	(0.889 - 1.891)	(0.931 - 2.788)	(0.932 - 2.005)	(0.959 - 2.636)
High school	1.569**	1.913**	1.675***	1.890**
	(1.096 - 2.247)	(1.123 - 3.258)	(1.141 - 2.458)	(1.144 - 3.121)
Graduate/professional	2.135***	2.635***	2.302***	2.613***
	(1.384 - 3.291)	(1.517 - 4.577)	(1.512 - 3.506)	(1.543 - 4.424)
Parity (0 children reference)				
1 child	0.692	0.728	0.674*	0.729
	(0.443 - 1.081)	(0.459 - 1.156)	(0.424 - 1.073)	(0.472 - 1.124)
2 children	1.452*	1.514*	1.456*	1.522**
	(0.953 - 2.210)	(0.980 - 2.341)	(0.941 - 2.254)	(1.005 - 2.306)
3 children	1.175	1.168	1.180	1.168
	(0.815 - 1.695)	(0.811 - 1.682)	(0.809 - 1.721)	(0.826 - 1.651)
4 children	1.141	1.131	1.120	1.139
	(0.706 - 1.845)	(0.697 - 1.835)	(0.680 - 1.845)	(0.706 - 1.837)
5 children	0.292***	0.315***	0.286***	0.314***
	(0.179 - 0.478)	(0.187 - 0.528)	(0.174 - 0.468)	(0.188 - 0.526)
Buddhist	2.605***	2.543***	2.793***	2.906***
	(1.731 - 3.921)	(1.743 - 3.711)	(1.731 - 4.506)	(1.836 - 4.601)
Constant	0.986	2.534**	1.253	2.399*
	(0.201 - 4.836)	(1.041 - 6.166)	(0.417 - 3.764)	(0.920 - 6.255)
Observations	2389	2389	2389	2389

Robust cieform in parentheses

*** *p* < 0.01, ** *p* < 0.05, * *p* < 0.1

Model 2 tested the association between the duration of the reproductive health program and modern method use. Women living in townships where the reproductive health program had been in existence for at least 10 years had 1.428 higher odds of reporting modern method use compared to women who reported living in townships where the programs had existed for less than 10 years [CI: 1.016-2.008]. In this model, younger age, higher education, and being Buddhist was also associated with use of modern method.

Model 3 tested the association between the number of IUD providers living in a township and modern method use. Compared to townships with no providers, townships with one provider had 3.647 higher odds of using a modern method and townships with two or more providers had 2.667 higher odds of using a modern method. Both were statistically significant (*p* < 0.001), even after adjusting for township-level effects and demographic characteristics. In this model, young ages, higher education levels, and being Buddhist was associated with modern method use.

Lastly, model 4 tested the association between the number of reproductive health providers that belonged to the PSI franchise network and use of modern method. Women who live in townships with 6-12 providers 1.519 times as likely to use a modern method compared to women living in townships with less than 6 providers [CI: 0.972-2.375], although this was only marginally statically significant (*p* < 0.1). Similarly, women who lived in townships with 13 or more providers had 1.458 higher odds of using a modern method compared to women who lived in townships with less than 6 providers [CI: 0.945-2.249] (marginally statistically significant at *p* < 0.1). Similar to other models, younger age, higher education status, and being Buddhist was associated with higher odds of using a modern method of contraception.

## Discussion

This study found that there was a positive association between social franchise programs and modern contraceptive use. Approximately 60% of women in this study used a modern method of contraception compared to data from a nationally-representative sample in 2009 that suggests that only 46% of ever married women used family planning [2]. Moreover, this study also assessed the dose-response and intensity of the program by examining the duration of the program and numbers of providers in each township. The data showed that use of modern contraception was higher among women living in townships where the social franchise program operated longer as well as among women living in townships with higher number of social franchised providers.

Furthermore, this study found differences in PSI/M’s reproductive health program (RH) vs. IUD program. While modern contraceptive use increased with the longer duration of the program for both the RH and IUD program, the effect size was higher for the IUD program. This further corroborates the findings that social franchise programs increase modern contraceptive use because the IUD program is considered to be more intensive and comprehensive compared to the RH-only program. First, the IUD programs recruited clinics that had a certain level of skill. The IUD program also included community public awareness campaigns regarding the IUD, as well as RH more broadly. In addition to providing RH services, including IUDs, the program also included a component in which Reproductive Health Promoters would bring women into the clinics and support them through the process of having an IUD inserted. Although we were unable to test this directly, one potential explanation for increased overall contraceptive use may be attributed to community health worker’s education around acceptance and knowledge of family planning at the township level. Further work should explore potential explanations for these findings. The study found that the odds of using a modern contraceptive method increased more with the duration IUD program and number of IUD program’s providers than that of RH program. While the study was not able to assess the impact of community and educational campaigns, it can be hypothesized that increasing community-level awareness and method choice increased contraceptive use at the township level.

Moreover, the study also found that more than 40% of women were accessing their modern contraceptive methods from the private sector. While this study was not able to assess whether the private sector was specifically a franchised clinic, this finding is important for future program planning for reproductive health services. Traditionally, modern contraceptive methods, except for condoms and pills, were largely available in the public sector. A significant portion of women in both rural and urban areas went to the private sector, particularly for IUDs.

There are a number of limitations to this study. First, because there is no comparison group, our ability to assess the impact of the social franchise program on family planning is limited. This study is not able to assess whether contraceptive prevalence would have increased in these townships even without the program. Contraceptive use may have naturally increased due to other interventions, programs, or increased community knowledge and acceptance of family planning methods. However, this study attempted to account for this limitation by including the duration that reproductive health services and the IUD program has existed in each township as well as the number of providers. Therefore, the study was able to assess both the intensity and dose-response of the intervention.

Second, social franchise program measures, including duration of the program and number of clinics, were at the township level. There was an assumption that all providers started the reproductive health program and IUD program at the same time; however, this may not have been the case as providers may have been recruited at different time points. Other information about the social franchise program, such as quality of providers or location of clinics, would have also been useful in assessing the impact of the program on family planning use.

Finally, although this study includes information on the source of family planning method, we are unable to distinguish between franchised clinics from other private clinics. It is uncertain what percentage of women actually received their family planning method from a PSI franchise clinic. In many contexts, PSI will engage private clinics but do not brand the clinic as a franchise program; therefore, women may not know that they are attending a franchise clinic. Despite this, approximately 40% of women indicated that they received their most recent method from a private sector outlet, highlighting the importance of the private sector in future family planning programs.

Despite these limitations, this is the first study to examine the role of social franchise programs, duration of the program, and intensity of the program on the use of modern contraceptive methods. Reproductive health data is critically limited in Myanmar. This study used a large, population-based dataset that included 25 townships and allowed for township-level analyses. Programmatic data allowed for assessing differences across RH-only program and RH + IUD programs.

A number of programmatic and policy recommendations result from this study. Overall, the study suggests that there should be increased engagement of the private sector in Myanmar, as approximately 40% of women are receiving their family planning method from private hospitals and clinics. In particular, the private sector plays a particularly important role in the uptake of IUDs. Historically in Myanmar, only OBGYNS are able to insert IUDs in the public sector. PSI/Myanmar has trained a network of general practitioners (GPs) to also provide IUDs, a novel approach in the country. This increases the accessibility and availability of family planning methods, suggesting that this should perhaps be implemented at wider scale.

Social franchise programs engage private providers to not only provide modern contraceptive methods and increase choice of method, but also advertise different methods with the goal of increasing family planning knowledge and awareness. Therefore, social franchise programs show promise as a means to increase the availability of family planning, particularly in underserved areas of Myanmar. Moreover, study results indicate that IUD programs, in addition to the reproductive health program, are particularly influential in increasing family planning methods. Future reproductive health programs may want to integrate an IUD component in order to increase choice of method and overall method uptake.

## Conclusions

This study found evidence for an association between clinical social franchise programs and increased use of family planning methods. This study is important given the dearth of reproductive health information in Myanmar and clinical social franchise programs.

## Abbreviations

AMWs: Auxiliary midwives
GPs: General practitioners
IUD: Intrauterine device
MICS: Multiple indicator survey cluster
NGOs: Non-governmental organizations
PPS: Population size method
PSI/M: Population services international/Myanmar
RH: Reproductive health
SQH: Sun quality health
TB: Tuberculosis
